# A simple predictive model for puerperal infections: emphasizing risk factors and pathogen analysis

**DOI:** 10.3389/fcimb.2024.1464485

**Published:** 2024-12-12

**Authors:** Yanqing Wen, Xin Ming, Jing Yang, Hongbo Qi

**Affiliations:** ^1^ Department of Obstetrics and Gynecology, Women and Children’s Hospital of Chongqing Medical University, Chongqing, China; ^2^ Department of Obstetrics and Gynecology, Chongqing Health Center of Women and Children, Chongqing, China; ^3^ Department of Quality Management Section, Women and Children’s Hospital of Chongqing Medical University, Chongqing, China; ^4^ Department of Obstetrics and Gynecology, Guizhou Provincial People’s Hospital, Guizhou, China

**Keywords:** puerperal infection, pathogenic bacteria, drug sensitivity, nomogram, predictive model

## Abstract

**Background:**

Puerperal infection (PI) accounting for approximately 11% of maternal deaths globally is an important preventable cause of maternal morbidity and mortality. This study aims to analyze the high-risk factors and pathogenic bacteria of PI, design a nomogram to predict the risk of PI occurrence, and provide clinical guidance for prevention and treatment to improve maternal outcomes.

**Methods:**

A total of 525 pregnant women were included in the study. The mothers were randomly divided into a training cohort (n=367) and a test cohort (n=158). The performance of our model was assessed using the area under the receiver operating characteristic (ROC) curve, calibration curve, and decision curve analyses. All the women in the group of PI underwent blood culture tests, if the bacteria were detected, drug sensitivity tests were performed. The drug sensitivity spectrum was recorded and analyzed.

**Results:**

Univariate analysis showed that 12 indicators were significantly different (P < 0.05). Logistic regression analysis showed 6 factors, such as parity, number of vaginal examinations, amount of postpartum bleeding, antibiotics administered in one week before admission, induced labor, and indwelling catheter were significantly different between the PI group and control group (P < 0.05). The area under the ROC curve was 0.904 (95% CI: 0.871-0.936) in the training set and 0.890 (95% CI: 0.837-0.942) in the test set. The calibration curve of the nomogram showed good agreement between prediction and observation. The analysis of the clinical decision curve showed that the nomogram is of practical significance. There were 100 patients with positive blood cultures in the PI group, and *Escherichia.coli* was the main pathogenic bacteria, accounting for 89%. The sensitivity to Meropenem and Imipenem was 100%, to Piperacillin tazobactam 97.75%, to Ceftazidime 95.51%, and to Amoxicillin/Clavulanat (AMC) was 93.26%.

**Conclusion:**

The risk of PI will be significantly reduced by controlling the number of vaginal examinations less than 4 times, postpartum hemorrhage less than 414ml, and reducing the time of urethral catheter indwelling. If PI was clinically diagnosed or highly suspected, it was recommended to use antibiotics that were sensitive to *Escherichia. coli*, such as Piperacillin tazobactam, Ceftazidime, and AMC

## 1 Introduction

Puerperal infection (PI) is defined as a local or systemic infection resulting from pathogen invasion of the genital tract, occurring at any time during the 6-week postpartum period. It is a common complication of the puerperium, with an incidence of about 6% (Karsnitz, 2013; Tardieu and Schmidt, 2017; Li et al., 2023). PI is characterized by a brief incubation period, sudden onset, and rapid fluctuations in condition. In severe cases, it may evolve into sepsis, progressing to systemic inflammatory response syndrome and multiple organ dysfunction syndromes, thereby posing a significant threat to maternal well-being (Kobayashi et al., 2017; Li et al., 2023). It was estimated that at least 75,000 maternal deaths from puerperal sepsis occur each year, mostly in low-income countries. Even high-income countries reported relatively high incidences ranging from 0.1 to 0.6 per 1000 deliveries (van Dillen et al., 2010; Acosta et al., 2013). At present, there are many literature reports on PI caused by group A streptococcal infection, especially in developed countries, and it is the main pathogen of PI leading to maternal mortality (Acosta et al., 2014). However, in China, we have found very few cases of puerperal infections caused by group A streptococcal infections. At the same time, bacterial resistance to commonly used antibiotics is increasing at an alarming rate. Therefore, it is important to understand the pathogens of PI and their susceptible antimicrobial spectrum (Song et al., 2020).

Early identification of PI can prevent adverse maternal outcomes, decrease expenses, and enhance the quality of healthcare. However, there is a scarcity of visual forecasting models that can be utilized for PI. The nomogram model has been extensively employed as a calibrated visualization tool in clinical settings to predict various outcomes. This model is based on multivariate analysis and integrates the results of logistic or Cox regression to a great extent to predict the probability of a certain clinical event in patients along with intuitive graphical presentations. It offers clinicians a basis for developing more efficient and personalized treatment plans.

This study aimed to analyze the risk factors of PI and construct a simple and effective multi-factorial prevention model, hoping to provide a reference for the formulation of maternal clinical management rules and infection control strategies. Additionally, this study revealed the most common pathogens and described the sensitivity spectrum of antimicrobial drugs, thereby offering a reliable foundation for the selection of antimicrobial drugs for puerperal infection.

## 2 Materials and methods

### 2.1 Study population

This was a retrospective case-control study including 525 women who gave birth at CQHCWC between January 1, 2022 and September 30, 2023. CQHCWC is a tertiary Class A maternal and infant care center with approximately 20,000 deliveries per year, accepting pregnant women from all regions of Chongqing, as well as from northwest and southwest China, including Guizhou, Sichuan, Tibet, Hubei and other regions. The study population was divided into two groups, one was women who met the criteria for PI, concluding 262 cases, and the other was randomly selected pregnant women who were hospitalized at the same time and gave birth with non-puerperal infection, concluding 263 cases. The overall data is randomly divided into training set (n=367) and verification (n=158) set according to the ratio of 7:3. Throughout this procedure, we establish a random seed to guarantee the randomness and reproducibility of the sampling (Wu et al., 2021).

Pregnant women in the PI group need to meet the following criteria:

1. Time of onset: at the time of delivery or within 42 days postpartum.
2. Fever: temperature ≥38°C during labor or 42 days postpartum, 4-hour intervals, ≥2 times.
3. Clinical signs: abdominal, or uterine, or pelvic pain, or smelly discharge, or poor healing of the incision.
4. Laboratory test: white blood cell count (WBC) ≥15×10^9^/L and neutrophil percentage (N%) ≥ 85%.

Exclusion Criteria of PI group: Confirmed diagnosis of upper respiratory tract infection, urinary tract infection, mastitis.

The normal control group included pregnant women who had normal labor at the same time and had no symptoms of infection.

### 2.2 Methods

Hospital records for each pregnant woman were reviewed, and medical and obstetric data were extracted. The data collected were as follows:

1. Demographic data: age, weight, height, Body Mass Index (BMI), gestational age, parity, and gravidity.
2. Laboratory data: laboratory results at admission and onset of fever, including WBC, N %, hemoglobin (HGB), blood culture, and drug-sensitive tests, Group B Streptococcus (GBS), albumin.

All the patients in PI group underwent blood culture tests. Both aerobic and anaerobic cultures were performed simultaneously for each patient, with the cultures being incubated for five days. All the isolates were identified at the species level by the MALDI-TOF/TOF (Bruker, Germany), and routine antimicrobial susceptibility testing was performed by using the BD Phoenix system. At the same time, Escherichia coli ATCC25922 was selected as the standard strains for minimum inhibitory concentration (MIC) detection. All standard strains were purchased from American Type Culture Collection and all antibiotics were purchased from Meilunbio in China.

3. Complications of pregnancy: premature rupture of membranes (PROM), preterm premature rupture of membranes (PPROM), gestational diabetes mellitus (GDM), oligohydramnios, gemellary pregnancy, intrahepatic cholestasis of pregnancy (ICP), gestational hypertension, cervical cerclage, central placenta previa;
4. Perinatal conditions: delivery mode, number of vaginal examinations, amniotic fluid volume, amount of bleeding, antibiotics administered within one week before admission, induced labor, amniotic fluid color, residual placental tissue in the uterine cavity, suturing the uterine cavity to stop bleeding, uterine balloon tamponade, colpoperineal laceration, episiotomy, cervical laceration, forceps delivery, incision hematoma, B-Lynch uterine compression sutures, indwelling catheter.

### 2.3 Statistical analysis

We used Kolmogorov-Smirnov to test the normality of the continuous variables, and the skewed distribution was expressed as the median and quartile range. Count data were expressed as a rate (%). Clinical characteristics were compared using the Kruskal-Wallis Test (continuous variables) and χ^2^ test (categorical variables). LASSO regression was used for feature selection in the training dataset. The nomogram was established as a result of multivariate logistic regression model with Five-fold cross-validation. We utilized a multivariate logistic regression to identify independent predictors, which were subsequently employed in developing a nomogram for predicting the incidence of puerperal infection. Predictor lines were drawn upward to confirm the positioning of the points on the “Total Points” axis, followed by a downward projection onto the lower scales, thereby determining the likelihood of puerperal infection. The Hosmer–Lemeshow test was used to assess goodness of fit. The receiver operating characteristic curve, area under the ROC curve, concordance index, and calibration curve were used to evaluate the predictive accuracy and conformity of the model. The decision curve analysis (DCA) reflected the net benefit of the model for patients. Clinical effectiveness was evaluated by plotting the clinical decision curve. To evaluate both discrimination and calibration, bootstrapping with 1000 resamples was performed.

Statistical analysis was performed with R 4.4.1 software (R Statistical Computing Foundation, Vienna, Austria). All statistical tests were two-sided and a *P* value <0.05 was considered significant.

### 2.4 Ethical statement

This survey research was approved by the ethics committee of the Women and Children’s Health Hospital of Chongqing Medical University (CQHCWC).

## 3 Results

### 3.1 Demographic characteristics

From January 2022 to September 2023, a total of 25,666 pregnant women gave birth at CQHCWC. 525 mothers aged 18 to 41 years were included in the study. We divided the dataset into a training dataset and a test dataset according to 7:3 (367:158 cases). The average BMI of the enrolled mothers was 27.06 ± 3.22 kg/m^2^. Overall, 48.5% (178/367) of mothers in the training set and 53.2% (84/158) of mothers in the validation set had a puerperal infection. The p-values of all these factors exceed 0.05, which indicates that there were no statistically significant differences between the training dataset and the validation dataset as shown in **Supplementary Table S1**. **Table 1** shows the baseline characteristics of the normal group and puerperal infection group. Parity, albumin at admission, number of vaginal examinations, amniotic fluid volume, amount of bleeding, PROM, antibiotics administered within one week before admission, induced labor, amniotic fluid color, mode of delivery, suturing the uterine cavity during cesarean section to stop bleeding, and indwelling catheter were statistically significant between the two groups in the training dataset (*P* < 0.05).

**Table 1:** The baseline characteristics of datasets.

Variable	Training dataset			Validation dataset		
	Non-puerperal infection (n=189)	Puerperal infection (n=178)	P value	Non-puerperal infection (n=74)	Puerperal infection (n=84)	P value
Age	29.00 [27.00, 32.00]	29.00 [27.00, 31.00]	0.499	29.00 [27.00, 31.00]	28.50 [27.00, 31.00]	0.690
Weight	67.50 [63.00, 72.00]	67.00 [61.50, 71.50]	0.493	65.85 [60.12, 74.00]	68.00 [62.38, 75.00]	0.239
Height	159.00 [156.00, 162.00]	158.00 [155.00, 161.00]	0.088	159.00 [155.00, 162.00]	158.00 [155.00, 160.00]	0.245
BMI	26.81 [24.97, 28.46]	27.12 [24.62, 29.41]	0.680	26.52 [24.48, 28.56]	27.47 [25.14, 29.49]	0.084
Gestational week	39.14[38.29,39.86]	39.43[38.14,40.29]	0.184	39.07 [38.18, 40.00]	39.36 [38.00, 40.29]	0.573
Gravity	2.00[1.00,3.00]	1.00[1.00,2.00]	0.653	1.00 [1.00, 2.00]	2.00 [1.00, 2.00]	0.421
Parity	0.00[0.00,1.00]	0.00[0.00,0.00]	**<0.001**	0.00 [0.00, 0.00]	0.00 [0.00, 0.00]	0.065
WBC at admission (10^9/^L)	8.70 [7.30, 10.50]	8.80 [7.40, 10.57]	0.577	8.95 [7.43, 11.47]	9.30 [7.65, 11.22]	0.976
N% at admission	76.30 [73.50, 80.30]	76.95 [71.60, 80.45]	0.741	78.00 [72.10, 82.38]	77.25 [72.70, 81.38]	0.499
HGB at admission (10^9/^L)	121.00 [113.00, 129.00]	122.00 [113.00, 128.75]	0.876	124.00 [111.25, 129.75]	120.85 [112.75, 128.00]	0.308
Albumin at admission	37.00 [35.00, 38.00]	36.00 [34.00, 38.00]	**0.004**	37.00 [36.00, 38.75]	36.00 [34.00, 37.00]	**0.002**
Number of vaginal examinations	3.00 [1.00, 5.00]	5.00 [1.00, 8.00]	**<0.001**	4.00 [1.00, 5.00]	5.00 [1.00, 7.25]	**0.029**
Amniotic fluid volume	400.00 [300.00, 500.00]	300.00 [200.00, 500.00]	**<0.001**	400.00 [300.00, 500.00]	300.00 [200.00, 500.00]	0.056
Amount of bleeding (ml)	415.00 [331.00, 498.00]	402.50 [318.50, 490.00]	**<0.001**	325.50 [280.75, 413.75]	445.00 [395.00, 540.00]	**<0.001**
PROM						
No	149 (78.8)	101 (56.7)	**<0.001**	60 (81.1)	50 (59.5)	**0.006**
Yes	40 (21.2)	77 (43.3)		14 (18.9)	34 (40.5)	
GDM						
No	160 (84.7)	149 (83.7)	0.916	61 (82.4)	71 (84.5)	0.890
Yes	29 (15.3)	29 (16.3)		13 (17.6)	13 (15.5)	
GBS						
No	185 (97.9)	172 (96.6)	0.677	74 (100.0)	81 (96.4)	0.290
Yes	4 (2.1)	6 (3.4)		0 (0.0)	3 (3.6)	
Oligohydramnios						
No	179 (94.7)	165 (92.7)	0.562	70 (94.6)	81 (96.4)	0.864
Yes	10 (5.3)	13 (7.3)		4 (5.4)	3 (3.6)	
Gemellary pregnancy						
No	185 (97.9)	169 (94.9)	0.215	72 (97.3)	82 (97.6)	1.000
Yes	4 (2.1)	9 (5.1)		2 (2.7)	2 (2.4)	
ICP						
No	188 (99.5)	171 (96.1)	0.061	74 (100.0)	80 (95.2)	0.163
Yes	1 (0.5)	7 (3.9)		0 (0.0)	4 (4.8)	
Gestational hypertension						
No	183 (96.8)	176 (98.9)	0.324	69 (93.2)	82 (97.6)	0.344
Yes	6 (3.2)	2 (1.1)		5 (6.8)	2 (2.4)	
Cervical ligation						
No	189 (100.0)	177 (99.4)	0.976	74 (100.0)	84 (100.0)	NA
Yes	0 (0.0)	1 (0.6)				
Central placenta previa						
No	187 (98.9)	176 (98.9)	1.000	73 (98.6)	82 (97.6)	1.000
Yes	2 (1.1)	2 (1.1)		1 (1.4)	2 (2.4)	
Antibiotics administered within 1 week before admission						
No	188 (99.5)	129 (72.5)	**<0.001**	74 (100.0)	63 (75.0)	**<0.001**
Yes	1 (0.5)	49 (27.5)		0 (0.0)	21 (25.0)	
Induced labor						
No	159 (84.1)	92 (51.7)	**<0.001**	63 (85.1)	49 (58.3)	**<0.001**
Yes	30 (15.9)	86 (48.3)		11 (14.9)	35 (41.7)	
Amniotic fluid color						
0	151 (79.9)	120 (67.4)	**<0.001**	60 (81.1)	60 (71.4)	0.383
1	22 (11.6)	15 (8.4)		6 (8.1)	7 (8.3)	
2	13 (6.9)	26 (14.6)		7 (9.5)	13 (15.5)	
3	3 (1.6)	17 (9.6)		1 (1.4)	4 (4.8)	
Mode of labor						
Vaginal	100 (52.9)	40 (22.5)	**<0.001**	47 (63.5)	19 (22.6)	**<0.001**
Cesarean section (CS)	89 (47.1)	138 (77.5)		27 (36.5)	65 (77.4)	
Suturing the uterine cavity during CS to stop bleeding						
No	189 (100.0)	169 (94.9)	**0.005**	74 (100.0)	82 (97.6)	0.533
Yes	0 (0.0)	9 (5.1)		0 (0.0)	2 (2.4)	
Uterine balloon tamponade						
No	188 (99.5)	174 (97.8)	0.333	74 (100.0)	84 (100.0)	NA
Yes	1 (0.5)	4 (2.2)				
Bimanual examination						
No	189 (100.0)	177 (99.4)	0.976	74 (100.0)	84 (100.0)	NA
Yes	0 (0.0)	1 (0.6)				
Incision hematoma						
No	186 (98.4)	172 (96.6)	0.443	74 (100.0)	82 (97.6)	0.533
Yes	3 (1.6)	6 (3.4)		0 (0.0)	2 (2.4)	
Colpoperineal laceration						
No	170 (89.9)	166 (93.3)	0.341	63 (85.1)	79 (94.0)	0.112
Yes	19 (10.1)	12 (6.7)		11 (14.9)	5 (6.0)	
Episiotomy						
No	178 (94.2)	174 (97.8)	0.143	69 (93.2)	82 (97.6)	0.344
Yes	11 (5.8)	4 (2.2)		5 (6.8)	2 (2.4)	
Cervical laceration						
No	182 (96.3)	175 (98.3)	0.386	68 (91.9)	83 (98.8)	0.085
Yes	7 (3.7)	3 (1.7)		6 (8.1)	1 (1.2)	
Forceps delivery						
No	185 (97.9)	175 (98.3)	1.000	72 (97.3)	82 (97.6)	1.000
Yes	4 (2.1)	3 (1.7)		2 (2.7)	2 (2.4)	
B-Lynch uterine compression sutures						
No	187 (98.9)	174 (97.8)	0.627	74 (100.0)	82 (97.6)	0.533
Yes	2 (1.1)	4 (2.2)		0 (0.0)	2 (2.4)	
Indwelling catheter						
No	171 (90.5)	54 (30.3)	**<0.001**	71 (95.9)	26 (31.0)	**<0.001**
Yes	18 (9.5)	124 (69.7)		3 (4.1)	58 (69.0)	

Bold indicates statistically significant results P < 0.05.

### 3.2 Screening for predictive factors

**Figure 1:**
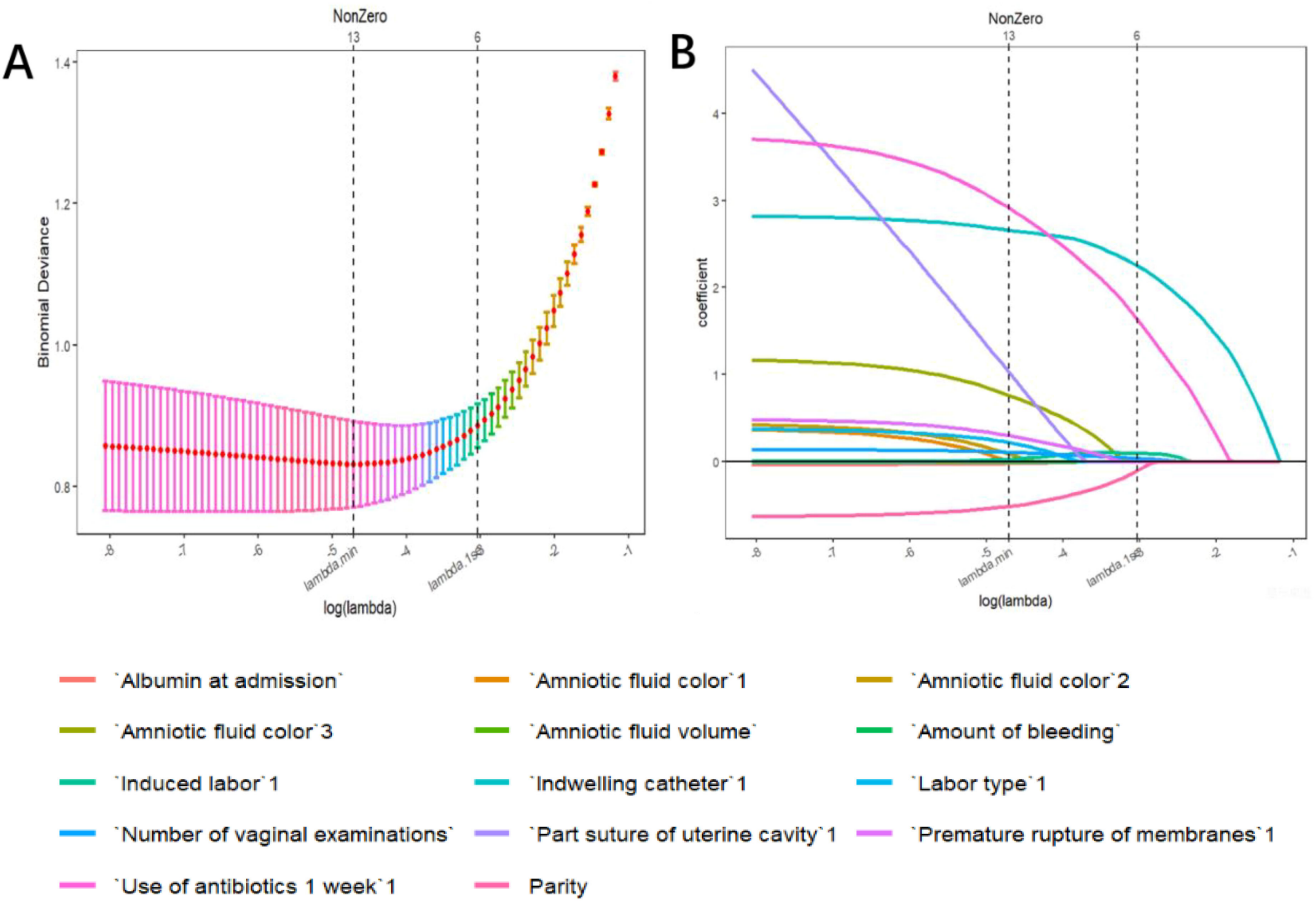
Variable selection by the LASSO binary logistic regression model. **(A)** Following verification of the optimal parameter (l) in the LASSO model, the mean squared error changes for the Log (l) value, and the vertical dotted line near Log (l) is drawn based on 1 standard error criteria. **(B)** Six variables with nonzero coefficients were selected by deriving the optimal lambda.

To prevent overfitting and ensure the stability of the model, the LASSO logistic regression model was ultimately used. The performance was optimal in the training dataset when λ was 0.007 (**Figures 1A, B**). A total of 12 features were screened, including parity, albumin at admission, number of vaginal examinations, amniotic fluid volume, amniotic fluid color, amount of bleeding, PROM, antibiotics administered in one week before admission, induced labor, suturing the uterine cavity during cesarean section to stop bleeding, mode of delivery, and indwelling catheter, which will be used to construct the predictive model.

### 3.3 Risk prediction nomogram development

**Figure 2:**
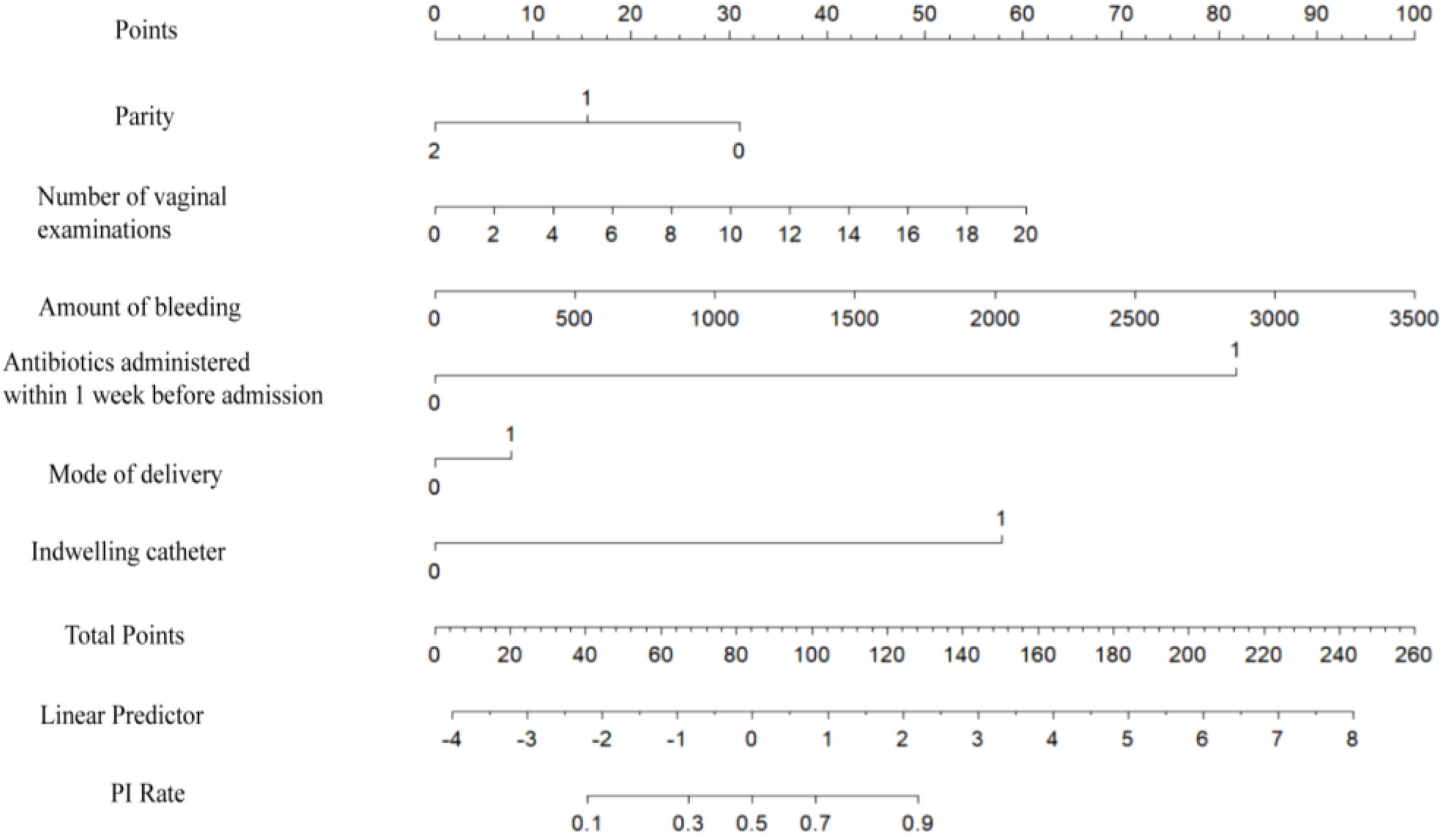
Nomogram to estimate the probability of PI. Find the predictor points on the uppermost point scale that correspond to each variable of the pregnant woman and add them up; the total points projected to the bottom scale indicate the probability of PI.

**Figure 3:**
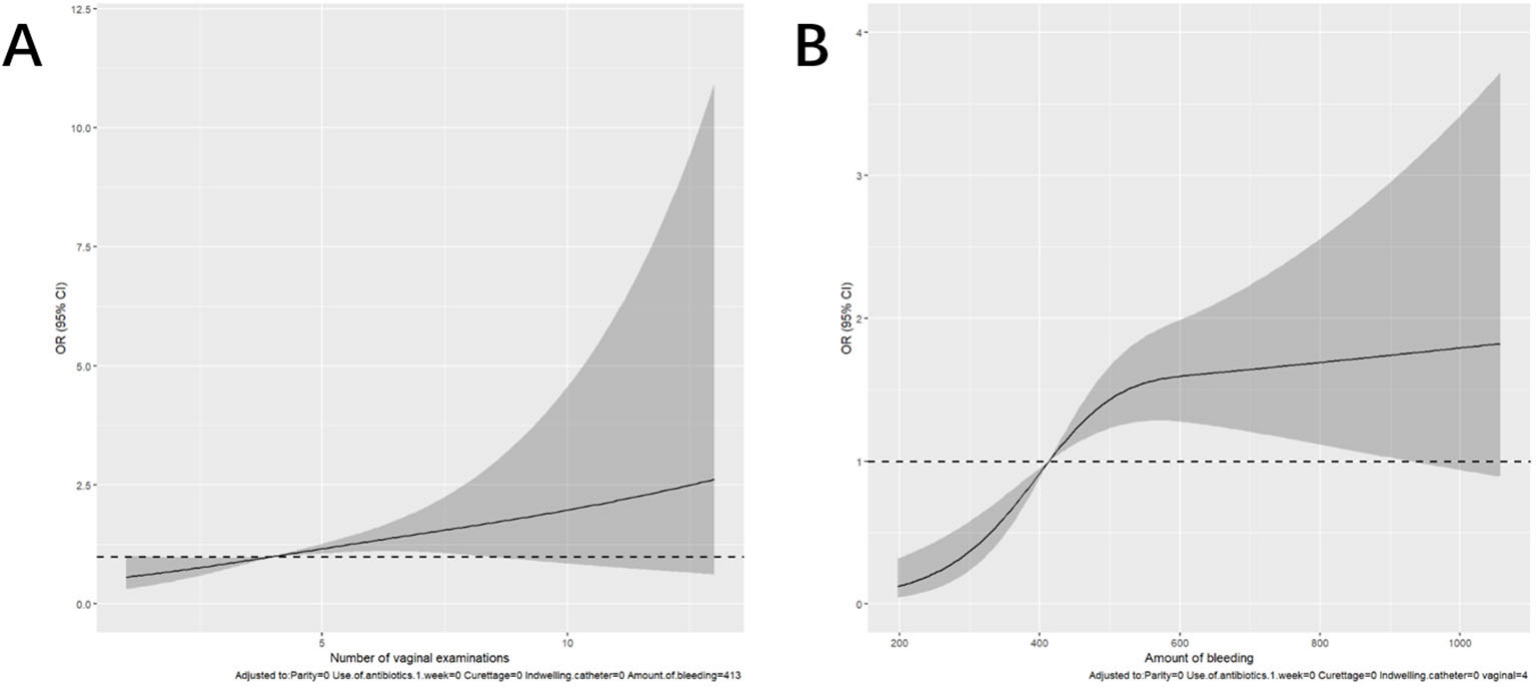
Restricted cubic spline (RCS) regression analysis. **(A)** The relationship between the number of vaginal examinations and PI. **(B)** The relationship between the amount of bleeding and PI.

The logistic regression model was constructed based on the above 12 factors. Parity (*OR*: 0.215, *95%CI*: 0.059-0.787), number of vaginal examinations (*OR*: 2.107, *95%CI*: 1.075-4.128), amount of bleeding (*OR*: 1.293, *95%CI*: 1.035-1.617), antibiotics administered in one week before admission (*OR*:61.303, *95%CI*: 7.491-501.71), induced labor (*OR*: 1.097, *95%CI*: 0.479-2.515), and indwelling catheter (*OR*: 21.726, *95%CI*: 11.010-42.874) were included in the multivariate logistic regression (**Table 2**). A nomogram for the diagnosis of PI was established based on the LASSO logistic regression model (**Figure 2**, C-index=0.905),Additionally, we visually deploy the predictive model on shinyapps.io (https://puerperalinfection.shinyapps.io/infection/). We further used RCS regression analysis to find out that the risk of PI increased when the number of vaginal examinations was more than 4 times (**Figure 3A**) and postpartum bleeding was greater than 414 ml (**Figure 3B**).

**Table 2:** Multivariable logistic model of the probability of puerperal infection in the training dataset.

Variables	*B*	*SE*	*P*	*OR*	*95%CI*	
					Low	High
Parity	-0.769	0.331	0.020	0.215	0.059	0.787
Number of vaginal examinations	0.124	0.057	0.030	2.107	1.075	4.128
Amount of bleeding	0.002	0.001	0.024	1.293	1.035	1.617
Antibiotics administered within 1 week before admission	4.116	1.073	<0.001	61.303	7.491	501.71
Induced labor	0.093	0.423	0.826	1.097	0.479	2.515
Indwelling catheter	3.079	0.347	<0.001	21.726	11.010	42.874

### 3.4 Model validation

**Figure 4:**
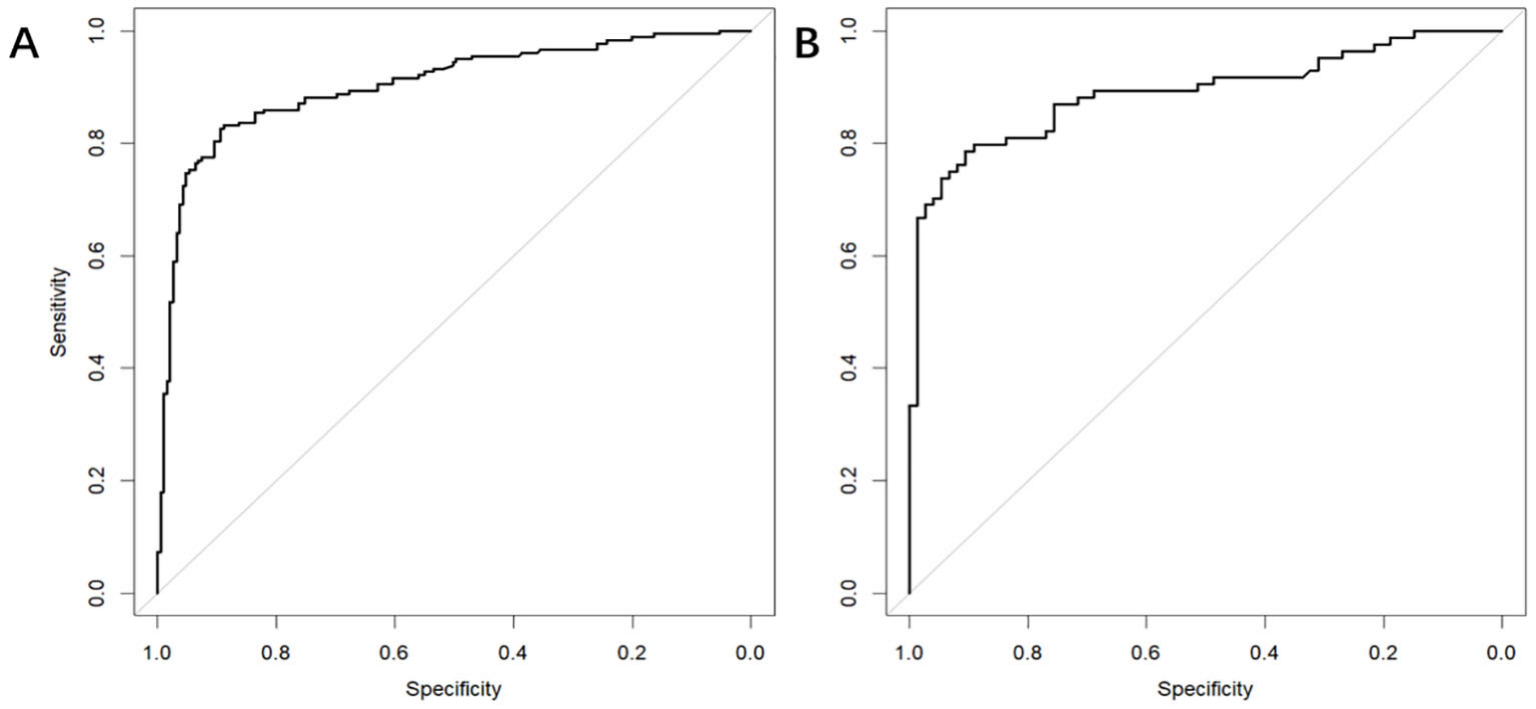
The receiver operating characteristic (ROC) curves of nomograms in the training and validation datasets, respectively. **(A)** The AUC value of the training dataset is 0.904, 95% CI: 0.871-0.936 (p < 0.05). **(B)** The AUC value of the validation dataset is 0.890, 95% CI: 0.837–0.942 (p < 0.05).

**Figure 5:**
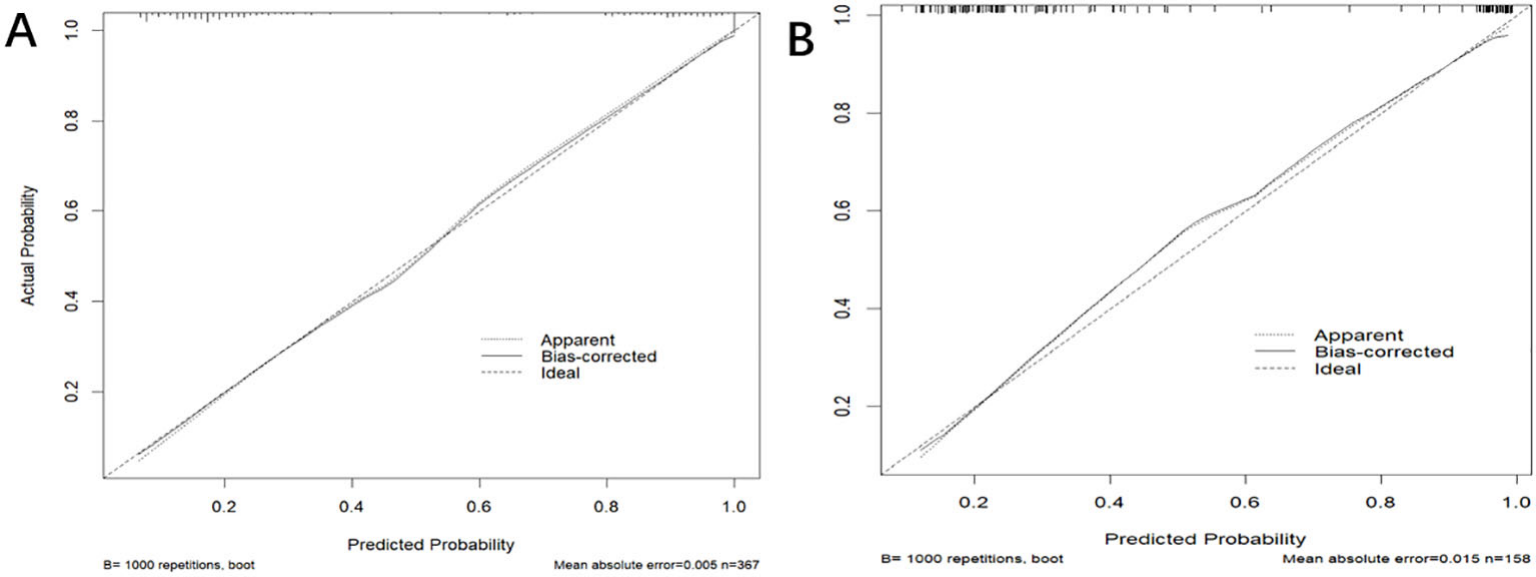
The calibration curve of the nomogram for predicting PI in the training dataset **(A)** and validation dataset **(B)**, respectively. Calibration focused on the accuracy of the probability between the predictive model and the actually observed value. The y-axis represents the actual diagnosed cases of puerperal infection, the x-axis represents the predicted risk of puerperal infection, and the solid line represents the prediction of the training dataset and the validation dataset **(B)**.

**Figure 6:**
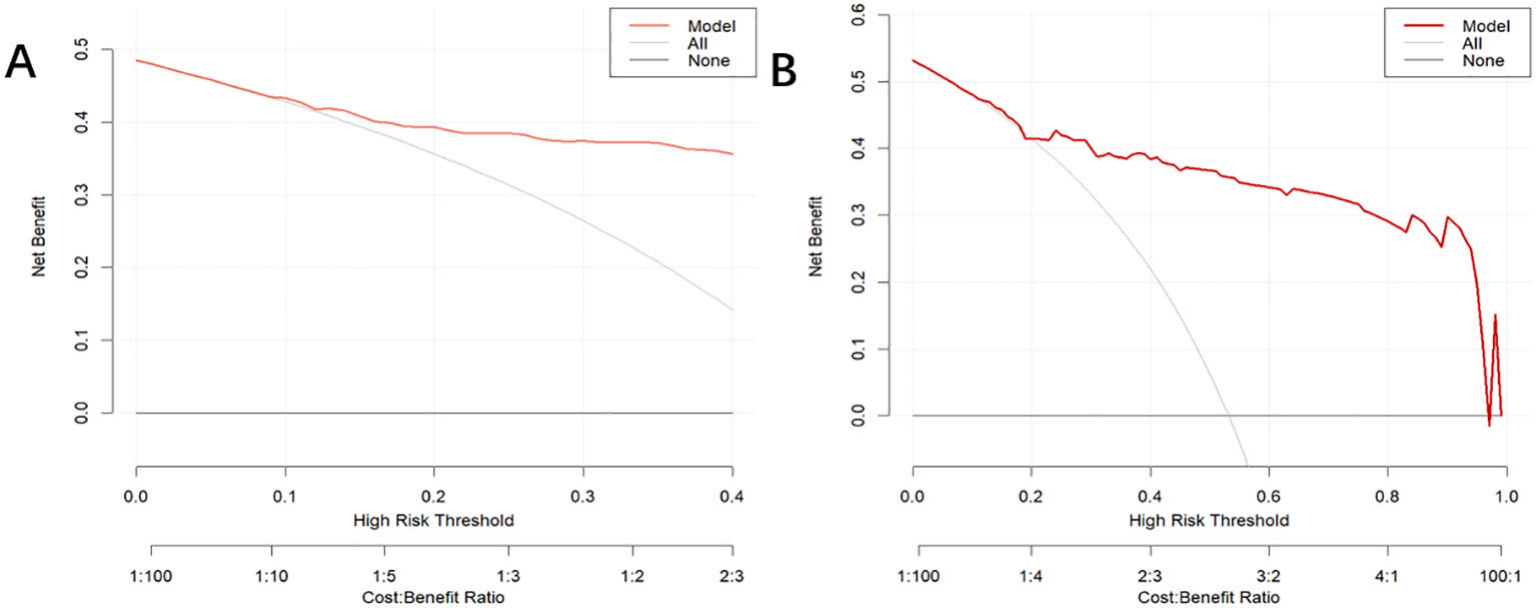
Decision curve analysis for the puerperal infection risk nomogram. The y-axis estimates the net benefit, the horizontal solid line represents the probability that the mother does not have a risk of puerperal infection, and the oblique solid line represents the probability of risk that the mother does not have a risk of puerperal infection. **(A)** Training dataset. **(B)** Validation dataset.

In the training dataset, the AUC was 0.904 (*95% CI*: 0.871-0.936) (**Figure 4A**), and the calibration curve was close to the ideal diagonal line (**Figure 5A**). Furthermore, the DCA showed significantly better net benefit in the predictive model (**Figure 6A**). Moreover, a validation dataset consisting of 158 mothers was used to test the accuracy of the nomogram. The AUC was 0.890 (*95% CI*: 0.837–0.942) (**Figure 4B**), reflecting a good accuracy of the nomogram. Meanwhile, the model had good consistency and the calibration curve of the validation cohort was also close to the ideal diagonal line (**Figure 5B**). Additionally, the DCA was employed to visually assess the clinical efficacy of the nomogram prediction model in both the training and validation datasets, as depicted in **Figure 6B**. The Clinical impact curves (**Supplementary Figure S1**) in the training and validation datasets are also well-fitted. This evaluation further confirms that the nomogram exhibits optimal predictive capability. The results indicate that the nomogram has a considerable potential for aiding clinical decision-making.

### 3.5 Drug resistance analysis

**Figure 7:**
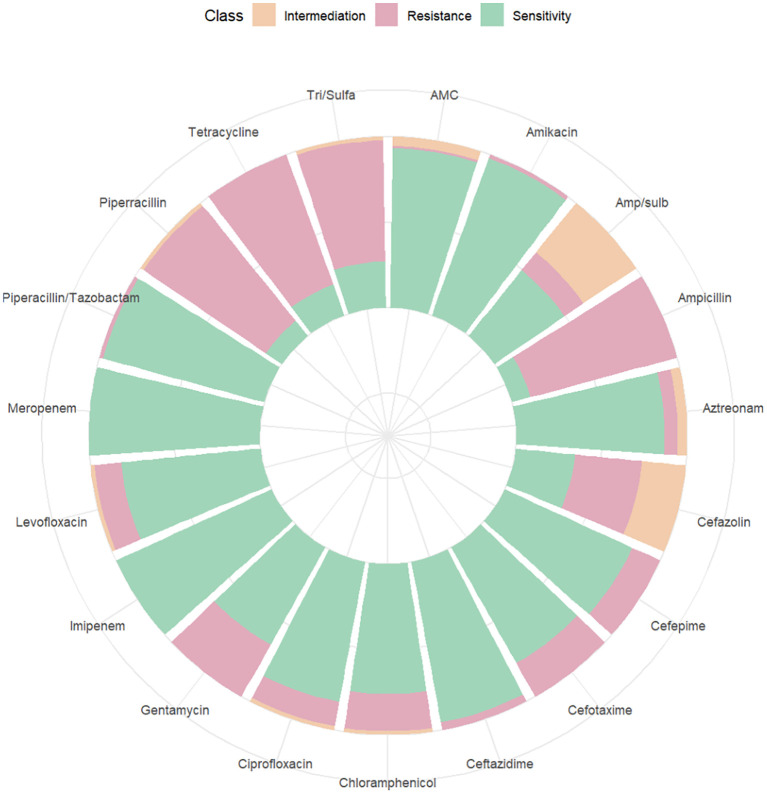
Drug sensitivity map of *Escherichia.coli*.

**Figure 8:**
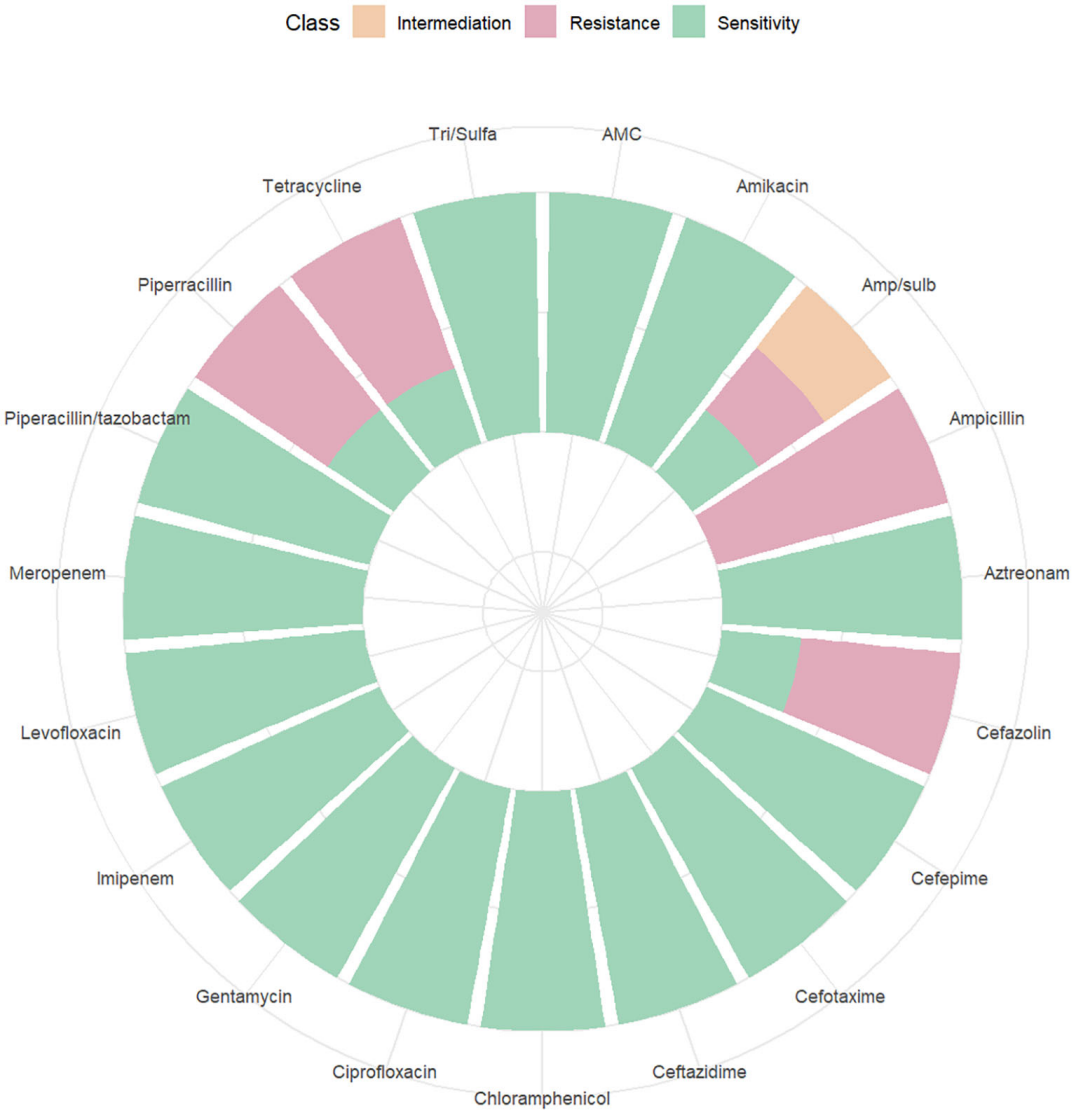
Drug sensitivity map of *Klebsiella pneumonia*.

Among the 262 PI women, 206 were full-term pregnant at term (≥ 37 weeks) and 42 were non-term pregnant women (< 37 weeks). The positive rate of blood culture in preterm pregnant women was higher than that in full-term pregnant women (*χ^2^* = 6.254, *P* = 0.012). 14 women were rehospitalized after discharge for PI (**Table 3**). The results of blood culture showed that 100 cases were positive, including 89 cases of *Escherichia.coli* (89% of positive blood culture), 3 cases with *Klebsiella pneumoniae*, 2 cases of *Staphylococcus epidermidis* and *Staphylococcus capitis*, and 1 case for each of *Burkholderia cepacia*, *Streptococcus agalactiae*, *Enterococcus faecalis*, and *Streptococcus mitis*. A drug susceptibility test was performed in patients with positive blood cultures. We found that the susceptibility of *Escherichia. coli* to Imipenem and Meropenem was 100%, to Piperacillin/tazobactam was 97.75%, and to Ceftazidime was 95.51% (**Figure 7**). The susceptibility of *Klebsiella pneumoniae* to Imipenem, Meropenem, Piperacillin/tazobactam, and Ceftazidime was 100% (**Figure 8**).

**Table 3:** Gestational week and blood culture distribution of puerperal infection group.

The time of onset of fever	Number	Positive blood culture	Negative blood culture	χ^2^	P value
≥37week	206	75	131	6.254	0.012
<37week	42	24	18		
Fever recurred after discharge	14	1	13		

## 4 Discussion

This study has important implications for maternal perinatal health. In the past, due to the lack of good predictive models, obstetricians often started to treat the infection at the onset of PI, and delayed treatment caused great trauma to the mother. On the other hand, some obstetricians used antibiotics without instructions for fear of PI, which increased the risk of drug resistance. To solve this challenging clinical dilemma, we developed a simplified and practical PI prediction model that utilizes LASSO and multivariate regression analyses to select six variables closely associated with PI. These risk factors for PI were parity, number of vaginal examinations, amount of bleeding, antibiotics administered in one week before admission, induced labor, and indwelling catheter. Obstetricians can utilize this nomogram to easily evaluate the risk level and implement targeted prevention and control measures.

The multivariate regression analysis revealed that an increased number of vaginal examinations was an independent risk factor for maternal infection (*OR*: 2.107, *95%CI*: 1.075-4.128). We also observed that conducting vaginal examinations more than 4 times was associated with an increased risk of infection. Similar results were also reported in previous research (Ngonzi et al., 2018; Saeed et al., 2019; Ketema et al., 2020; Oyato et al., 2024). With the increase in a number of vaginal examinations (especially after the membrane is broken), the balance of female vaginal flora may be broken, resulting in weakened defense ability, and pathogenic bacteria may enter the uterine cavity, abdominal cavity, and blood through the cervix, reproductive tract, and wound surface (Oyato et al., 2024). Furthermore, our study showed that indwelling catheter was also a risk factor for PI, which may be because routine catheter indwelling after cesarean section leads to retrograde bacteria and increases the risk of infection. After a cesarean section, the mother should be encouraged to get out of bed in advance and remove the catheter as soon as possible to reduce the risk of infection (Mackeen et al., 2024). Additionally, our study showed that there was an increasing incidence of PI associated with postpartum hemorrhage, and the risk of puerperal infection was elevated when postpartum bleeding exceeded 414 ml. Postpartum hemorrhage causes damage to the maternal reproductive system to a certain extent, resulting in the balance of the body system being broken, and eventually leading to infection (Gonzalez-Brown and Schneider, 2020). After the occurrence of maternal bleeding, timely intervention measures should be taken to control the amount of blood loss within the safe line and minimize the impact of postpartum hemorrhage on the balance of the immune system, thereby reducing the risk of infection. Interestingly, antibiotics administered in 1 week before admission was also an independent risk factor for puerperal infection (Adeyemo et al., 2022). In addition, Miller et al. found that exposure to antibiotics during pregnancy has also been associated with an increased risk of hospitalization infections in children (Miller et al., 2018). Patients who were given antibiotics before delivery had pre-existing infections or had high-risk factors for infection. This might be the reason why these patients were at high risk of PI. In these patients, antibiotic choice during and after delivery or the puerperium was important. At present, *Escherichia*. *coli* is still the main pathogenic bacteria in China, which differs from developed countries. Here we recommended choosing antibiotics that are sensitive to *Escherichia coli*. Our research has found that multiparous women have a lower risk of puerperal infections than primiparous women. This may be due to the shorter duration of labor and the less vaginal examinations in multiparous women.

In the univariate analysis, we found that the CS rate was significantly higher in the PI group than in the control group in both the training and validation sets. The CS rate of all infected patients was 77.5%, of which 11 (4.2%) had bleeding from the placental attachment site, and repeated surgical procedures such as uterine sutures were performed during CS to stop the bleeding. CS and repeated uterine sutures, especially after prolonged labor or induced labor, increased the risk of puerperal infection. This was consistent with previous literature reports. A study from Canada reported that prolonged labor followed by a cesarean section increased the incidence of postpartum sepsis to as high as 30% (Ross et al., 2013). Leth et al. found that the risk of PI seems to be nearly 5-fold increased after cesarean section compared with vaginal birth (Leth et al., 2009). Also, we found that the infected group had lower amniotic fluid volume than the control group, suggesting that sufficient amniotic fluid may be a protective factor for puerperal infection. PROM might also be one of the reasons for the low volume of amniotic fluid in the infected group. In univariate analysis, PROM was also a risk factor for puerperal infection. PROM increases the risk of retrograde entry into the uterine cavity and is a well-established risk factor for infection. Our study demonstrated that lower levels of albumin upon admission may serve as a potential risk factor for postpartum infection. Albumin plays a pivotal role in both wound healing and immune function (Jiang et al., 2022), thereby suggesting that the association between preoperative hypoalbuminemia and puerperal infection is likely to be multifaceted. One plausible explanation for this intricate relationship could be attributed to the fact that mothers with diminished albumin levels might experience compromised nutritional status along with deficiencies in other essential vitamins and nutrients, leading to increased maternal susceptibility to pathogenic bacteria (Kishawi et al., 2020).

Pathogens are the main culprits responsible for PI, and choosing the right antimicrobial agent is critical to PI outcomes. We found that the main pathogen of PI was *Escherichia.coli*, and we analyzed its antimicrobial susceptibility. The result showed that its sensitivity to carbapenems (Meropenem and Imipenem) were 100%, to the beta-lactam compound drug such as Piperacillin Tazobactam and ceftazidime were over 95%. However, *Escherichia. coli* was less than 50% sensitive to ampicillin-tazobactam and only 11% sensitive to ampicillin. Therefore, prompt antibiotic change should be considered in pregnant women with high fever and suspected sepsis, or with CS after prolonged induction of labor, or with antibiotics administered within one week before termination of pregnancy, or with perioperative fever, and Piperacillin Tazobactam and Ceftazidime were the good choices.

Our nomogram demonstrated a significant level of discrimination (C-index = 0.905), and the decision curve analysis revealed a significantly improved net benefit in the predictive model. This model can be easily recalibrated for individual settings provided that the six predictor variables are available. However, our study was limited to a retrospective single-center and non-randomized controlled study. More prospective, multicenter studies are needed to increase the reliability of the extrapolation of results. In addition, the data on puerperal infection were not completely obtained as they may be referred to community hospitals. Further external validation is needed to optimize and improve the nomogram.

## 5 Conclusion

In conclusion, a nomogram that included 6 variables was developed to predict PI risk based on a LASSO and logistic regression analysis, which showed a good performance through internal validation. Based on scoring using the nomogram, we can effectively manage risk factors and implement targeted active surveillance promptly to reduce the occurrence of PI, and decrease costs related to prevention and control. All these indicate that we should strengthen the management of high-risk pregnancies, reduce the frequency of vaginal examination, reduce the amount of postpartum bleeding, and reduce the rate of CS. Once PI occurs, sepsis should be recognized as soon as possible. In addition, antibiotics should be carefully and rationally selected in the clinic to ensure the safety of drug use in patients while improving the therapeutic effect.

## Data Availability

The raw data supporting the conclusions of this article will be made available by the authors, without undue reservation.

## References

[B1] Acosta C. D. Knight M. Lee H. C. Kurinczuk J. J. Gould J. B. Lyndon A. (2013). The continuum of maternal sepsis severity: incidence and risk factors in a population-based cohort study. PloS One 8, e67175. doi: 10.1371/journal.pone.0067175 23843991 PMC3699572

[B2] Acosta C. D. Kurinczuk J. J. Lucas D. N. Tuffnell D. J. Sellers S. Knight M.. (2014). Severe maternal sepsis in the UK, 2011-2012: a national case-control study. PloS Med. 11, e1001672. doi: 10.1371/journal.pmed.1001672 25003759 PMC4086731

[B3] Adeyemo M. Oyeneyin L. Irinyenikan T. Gbala M. Akadiri O. Bakare B.. (2022). Pre-caesarean section vaginal preparation with chlorhexidine solution in preventing puerperal infectious morbidities: A randomized controlled trial. West Afr J. Med. 39, 369–374. 35489037

[B4] Gonzalez-Brown V. Schneider P. (2020). Prevention of postpartum hemorrhage. Semin. Fetal Neonatal Med. 25, 101129. doi: 10.1016/j.siny.2020.101129 32782215

[B5] Jiang B. Xu H. Xie J. Wang D. Gan Q. Zhou Z. (2022). Are the preoperative albumin levels and the albumin to fibrinogen ratio the risk factors for acute infection after primary total joint arthroplasty? Front. Surg. 9. doi: 10.3389/fsurg.2022.1043242 PMC9852750 36684164

[B6] Karsnitz D. B. (2013). Puerperal infections of the genital tract: a clinical review. J. Midwif. Womens Health 58, 632–642. doi: 10.1111/jmwh.12119 24406036

[B7] Ketema D. B. Wagnew F. Assemie M. A. Ferede A. Alamneh A. A. Leshargie C. T.. (2020). Incidence and predictors of surgical site infection following cesarean section in North-west Ethiopia: a prospective cohort study. BMC Infect. Dis. 20, 902. doi: 10.1186/s12879-020-05640-0 33256630 PMC7708170

[B8] Kishawi D. Schwarzman G. Mejia A. Hussain A. K. Gonzalez M. H. (2020). Low preoperative albumin levels predict adverse outcomes after total joint arthroplasty. J. Bone Joint Surg. Am. 102, 889–895. doi: 10.2106/JBJS.19.00511 32079884

[B9] Kobayashi N. Ahmed S. Sumi A. Urushibara N. Kawaguchiya M. Aung M. S. (2017). Collaborative research on puerperal infections in Bangladesh. Nihon Eiseigaku Zasshi 72, 106–111. doi: 10.1265/jjh.72.106 28552890

[B10] Leth R. A. Moller J. K. Thomsen R. W. Uldbjerg N. Norgaard M. (2009). Risk of selected postpartum infections after cesarean section compared with vaginal birth: a five-year cohort study of 32,468 women. Acta Obstet. Gynecol. Scand. 88, 976–983. doi: 10.1080/00016340903147405 19642043

[B11] Li P. Li Y. Zhang Y. Zhao L. Li X. Bao J.. (2023). Incidence, temporal trends and risk factors of puerperal infection in Mainland China: a meta-analysis of epidemiological studies from recent decade, (2010-2020). BMC Pregnancy Childbirth 23, 815. doi: 10.1186/s12884-023-06135-x 37996780 PMC10666378

[B12] Mackeen A. D. Sullivan M. V. Berghella V. (2024). Evidence-based cesarean delivery: preoperative management (part 7). Am. J. Obstet. Gynecol. MFM 6, 101362. doi: 10.1016/j.ajogmf.2024.101362 38574855

[B13] Miller J. E. Wu C. Pedersen L. H. de Klerk N. Olsen J. Burgner D. P. (2018). Maternal antibiotic exposure during pregnancy and hospitalization with infection in offspring: a population-based cohort study. Int. J. Epidemiol. 47, 561–571. doi: 10.1093/ije/dyx272 29415232

[B14] Ngonzi J. Bebell L. M. Fajardo Y. Boatin A. A. Siedner M. J. Bassett I. V.. (2018). Incidence of postpartum infection, outcomes and associated risk factors at Mbarara regional referral hospital in Uganda. BMC Pregnancy Childbirth 18, 270. doi: 10.1186/s12884-018-1891-1 29954356 PMC6022296

[B15] Oyato B. T. Debele T. Edosa D. Abasimel H. Z. Awol M. Kebede E. T.. (2024). Determinants of puerperal sepsis among postpartum women: a case-control study in East Shoa Zone public hospitals, Central Ethiopia. BMJ Open 14, e083230. doi: 10.1136/bmjopen-2023-083230 PMC11328626 38908838

[B16] Ross L. E. Grigoriadis S. Mamisashvili L. Vonderporten E. H. Roerecke M. Rehm J.. (2013). Selected pregnancy and delivery outcomes after exposure to antidepressant medication: a systematic review and meta-analysis. JAMA Psychiatry 70, 436–443. doi: 10.1001/jamapsychiatry.2013.684 23446732

[B17] Saeed K. B. Corcoran P. O’Riordan M. Greene R. A. (2019). Risk factors for surgical site infection after cesarean delivery: A case-control study. Am. J. Infect. Control 47, 164–169. doi: 10.1016/j.ajic.2018.07.023 30253904

[B18] Song H. Hu K. Du X. Zhang J. Zhao S. (2020). Risk factors, changes in serum inflammatory factors, and clinical prevention and control measures for puerperal infection. J. Clin. Lab. Anal. 34, e23047. doi: 10.1002/jcla.23047 31883276 PMC7083398

[B19] Tardieu S. C. Schmidt E. (2017). Group A streptococcus septic shock after surgical abortion: A case report and review of the literature. Case Rep. Obstet. Gynecol. 2017, 6316739. doi: 10.1155/2017/6316739 29085686 PMC5612605

[B20] van Dillen J. Zwart J. Schutte J. van Roosmalen J. (2010). Maternal sepsis: epidemiology, etiology and outcome. Curr. Opin. Infect. Dis. 23, 249–254. doi: 10.1097/QCO.0b013e328339257c 20375891

[B21] Wu W. T. Li Y. J. Feng A. Z. Li L. Huang T. Xu A. D.. (2021). Data mining in clinical big data: the frequently used databases, steps, and methodological models. Mil Med. Res. 8, 44. doi: 10.1186/s40779-021-00338-z 34380547 PMC8356424

