# Supplementary material for: A simple predictive model for puerperal infections: emphasizing risk factors and pathogen analysis
Source: Front Cell Infect Microbiol. 2024 Dec 12;14:1464485. doi: 10.3389/fcimb.2024.1464485 (PMC11669357; doi:10.3389/fcimb.2024.1464485)
Supplement: Supplementary file 1 [file Table1.docx]

Table S1. A comprehensive summary of the variables categorized by training and validation dataset in this study.

| Variable | | Training dataset (n=367) | Validation dataset (n=158) | *P* value |
| --- | --- | --- | --- | --- |
| Age |  | 29.00 [27.00, 31.00] | 29.00 [27.00, 31.00] | 0.855 |
| Weight |  | 67.00 [62.75, 72.00] | 67.75 [62.00, 74.00] | 0.621 |
| Height |  | 158.00 [155.00, 162.00] | 158.00 [155.00, 161.00] | 0.646 |
| BMI |  | 26.84 [24.72, 29.12] | 27.00 [24.99, 29.08] | 0.534 |
| Gestational week |  | 39.29 [38.29, 40.14] | 39.21 [38.04, 40.25] | 0.939 |
| Gravity |  | 2.00 [1.00, 3.00] | 2.00 [1.00, 2.00] | 0.838 |
| Parity |  | 0.00 [0.00, 0.00] | 0.00 [0.00, 0.00] | 0.089 |
| WBC at admission (10^9^ /L) |  | 8.80 [7.30, 10.50] | 9.15 [7.50, 11.40] | 0.117 |
| N% at admission |  | 76.70 [72.70, 80.30] | 77.40 [72.10, 82.02] | 0.200 |
| HGB at admission (10^9^ /L) |  | 121.00 [113.00, 129.00] | 121.00 [112.00, 129.00] | 0.725 |
| Albumin at admission |  | 36.00 [35.00, 38.00] | 36.00 [35.00, 38.00] | 0.352 |
| Number of vaginal examinations | | 4.00 [1.00, 7.00] | 4.00 [1.00, 7.00] | 0.294 |
| Amniotic fluid volume |  | 400.00 [300.00, 500.00] | 400.00 [300.00, 500.00] | 0.957 |
| Amount of bleeding (ml) |  | 415.00 [331.00, 498.00] | 402.50 [318.50, 490.00] | 0.378 |
| PI | No | 189 ( 51.5) | 74 ( 46.8) | 0.376 |
|  | Yes | 178 ( 48.5) | 84 ( 53.2) |  |
| PROM | No | 250 ( 68.1) | 110 ( 69.6) | 0.813 |
|  | Yes | 117 ( 31.9) | 48 ( 30.4) |  |
| GDM | No | 309 ( 84.2) | 132 ( 83.5) | 0.954 |
|  | Yes | 58 ( 15.8) | 26 ( 16.5) |  |
| GBS | No | 357 ( 97.3) | 155 ( 98.1) | 0.801 |
|  | Yes | 10 ( 2.7) | 3 ( 1.9) |  |
| Oligohydramnios | No | 344 ( 93.7) | 151 ( 95.6) | 0.531 |
|  | Yes | 23 ( 6.3) | 7 (4.4) |  |
| Gemellary pregnancy | No | 354 ( 96.5) | 154 ( 97.5) | 0.740 |
|  | Yes | 13 ( 3.5) | 4 ( 2.5) |  |
| ICP | No | 359 ( 97.8) | 154 ( 97.5) | 1.000 |
|  | Yes | 8 ( 2.2) | 4 ( 2.5) |  |
| Gestational hypertension | No | 359 ( 97.8) | 151 ( 95.6) | 0.257 |
|  | Yes | 8 ( 2.2) | 7 ( 4.4) |  |
| Cervical ligation | No | 366 ( 99.7) | 158 (100.0) | 1.000 |
|  | Yes | 1 ( 0.3) | 0 ( 0.0) |  |
| Central placenta previa | No | 363 ( 98.9) | 155 ( 98.1) | 0.744 |
|  | Yes | 4 ( 1.1) | 3 ( 1.9) |  |
| Antibiotics admitted within 1 week before admission | No | 317 ( 86.4) | 137 ( 86.7) | 1.000 |
|  | Yes | 50 ( 13.6) | 21 ( 13.3) |  |
| Induced labor | No | 251 ( 68.4) | 112 ( 70.9) | 0.642 |
|  | Yes | 116 ( 31.6) | 46 ( 29.1) |  |
| Amniotic fluid color | 0 | 270 ( 73.8) | 119 ( 75.8) | 0.560 |
|  | 1 | 37 ( 10.1) | 13 ( 8.3) |  |
|  | 2 | 39 ( 10.7) | 20 ( 12.7) |  |
|  | 3 | 20 ( 5.5) | 5 ( 3.2) |  |
| Mode of delivery | Vaginal | 140 ( 38.1) | 66 ( 41.8) | 0.495 |
|  | Cesarean | 227 ( 61.9) | 92 ( 58.2) |  |
| Suturing the uterine cavity during CS to stop bleeding | No | 358 ( 97.5) | 156 ( 98.7) | 0.590 |
|  | Yes | 9 ( 2.5) | 2 ( 1.3) |  |
| Uterine balloon tamponade | No | 362 ( 98.6) | 158 (100.0) | 0.325 |
|  | Yes | 5 ( 1.4) | 0 ( 0.0) |  |
| Bimanual examination | No | 366 ( 99.7) | 158 (100.0) | 1.000 |
|  | Yes | 1 ( 0.3) | 0 ( 0.0) |  |
| Incision hematoma | No | 358 ( 97.5) | 156 ( 98.7) | 0.590 |
|  | Yes | 9 ( 2.5) | 2 ( 1.3) |  |
| Colpoperineal laceration | No | 336 ( 91.6) | 142 ( 89.9) | 0.652 |
|  | Yes | 31 ( 8.4) | 16 ( 10.1) |  |
| Episiotomy | No | 352 ( 95.9) | 151 ( 95.6) | 1.000 |
|  | Yes | 15 ( 4.1) | 7 ( 4.4) |  |
| Cervical laceration | No | 357 ( 97.3) | 151 ( 95.6) | 0.457 |
|  | Yes | 10 (2.7) | 7 (4.4) |  |
| Forceps delivery | No | 360 ( 98.1) | 154 ( 97.5) | 0.900 |
|  | Yes | 7 ( 1.9) | 4 ( 2.5) |  |
| B-Lynch uterine compression sutures | No | 361 ( 98.4) | 156 ( 98.7) | 1.000 |
|  | Yes | 6 ( 1.6) | 2 ( 1.3) |  |
| Indwelling catheter | No | 225 ( 61.3) | 97 ( 61.4) | 1.000 |
|  | Yes | 142 ( 38.7) | 61 ( 38.6) |  |

Table S2 . The model coefficient with the smallest model deviation λ value

| Variable | λ min | λ se |
| --- | --- | --- |
| Parity | -0.5187 | -0.1086 |
| Number of vaginal examinations | 0.1127 | 0.0365 |
| Amount of bleeding | 0.0011 | 0.0001 |
| Antibiotics admitted within 1 week before admission | 3.0255 | 1.6336 |
| Induced labor | 0.0277 | 0.0943 |
| Indwelling catheter | 2.6977 | 2.2517 |

Note: λ min=0.007, λ se=0.048.


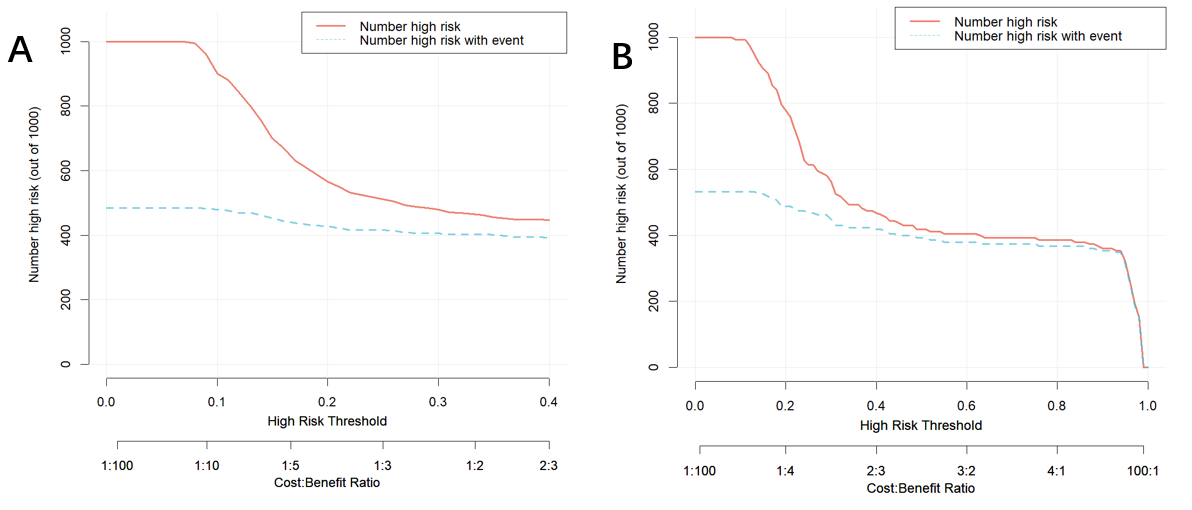


Fig S1. Clinical impact curve analysis for nomogram models. the red curve (number of high-risk mothers) indicates the number of mothers who are classified as puerperal infection (high risk) by the model at each threshold probability; the blue curve (number of high-risk mothers with the outcome) is the number of true puerperal infection at each threshold probability. (A) Training dataset. (B) Validation dataset.
